# EMT and Tumor Turning Point Analysis in 3D Spheroid Culture of HNSCC and Mesenchymal Stem Cells

**DOI:** 10.3390/biomedicines10123283

**Published:** 2022-12-19

**Authors:** Sabine Brylka, Florian Böhrnsen

**Affiliations:** Department of Oral and Maxillofacial Surgery, University Medicine Göttingen, 37075 Göttingen, Germany

**Keywords:** HNSCC, MSC, tumor spheroid culture, stroma-tumor spheroids, EMT, tumor microenvironment, tumor turning point

## Abstract

The prognosis, metastasis, and behavior of head and neck squamous cancer cells are influenced by numerous factors concerning the tumor microenvironment, intercellular communication, and epithelial-to-mesenchymal transition (EMT). The aim of this study was to examine the codependent interaction of the mesenchymal stroma with head and neck squamous cell carcinoma (HNSCC) in a 3D spheroid structure. To simulate stroma-rich and -poor 3D tumor microenvironments, cells of the established cell SCC-040 were cultured with human mesenchymal stromal cells (MSCs), forming 3D stroma-tumor spheroids (STSs). STSs were compared to uniform spheroids of SCC-040 and MSC, respectively. The expressions of CD24, β-catenin, SNAI2, and ZEB2 were analyzed via RT-qPCR. The immunohistochemical expressions of E-cadherin, connexin 43, vimentin, and emmprin were analyzed, and protein expression pathways as well as Akt signaling were assessed via protein analysis. A promotive effect on the expressions of EMT markers ZEB2 (*p* = 0.0099), SNAI2 (*p* = 0.0352), and β-catenin (*p* = 0.0031) was demonstrated in STSs, as was the expression of Akt pathway proteins mTOR (*p* = 0.007), Erk1/2 (*p* = 0.0045), and p70 S6 Kinase (*p* = 0.0016). Our study demonstrated a change in genetic expression patterns early on in tumor development, indicating a tumor turning point.

## 1. Introduction

Head and neck squamous cell carcinoma (HNSCC) results in an overall death rate of 35% worldwide. Caused mainly by smoking and alcohol abuse, the incidence of HNSCC is expected to continue to increase in the future as it remains a challenging disease, associated with physical and social disabilities [1,2]. Often, an initially benign change in cellular appearance might turn into a precancerous and later malignant tumor following an epithelial-to-mesenchymal transition (EMT). EMT, a process leading to a change in a cell’s phenotype, is characterized by the loss of the apical–basal polarity of epithelial cellular structures. During the process of EMT, epithelial cells lose their stationary function and gain mesenchymal characteristics, mobility, and the ability to invade surrounding tissue. The decompensation of cellular processes and overexpression of proteins promoting EMT (e.g., SNAI2 and ZEB2) can ultimately contribute to the proliferation and metastasizing of dedifferentiated epithelial cancer cells. Thus, EMT marks a crucial step in the progression and malignancy of cancerous diseases [3,4,5]. Because EMT is driven by growth factors such as TNF and interleukins as well as inter- and intracellular communication, the tumor microenvironment (TME) exerts a significant influence on tumor EMT [6]. Being composed of stromal cells, fibroblasts, endothelial cells, and immune cells, the TME and its crosstalk with the inert, dedifferentiated cancer cells lead to a codependent influence [7]. At first, cells of the TME try to contain the growth of the tumor by the secretion of cytokines such as TNF or INF-γ, trying to recruit immune and inflammatory cellular defense mechanisms. However, these mechanisms and continuous cell–cell interactions, activation, and stimulation of the TME often lead to a modulation of the TME to overcome the inhibitory signals [8]. The upregulation of EMT markers such as ZEB2, β-catenin, and SNAI2, as well as an upregulation of the Akt pathway and proteins such as Erk1/2 and S6 ribosomal protein, underline said modulation and promote the development of invasive characteristics. As a result, stromal cells of the TME can ultimately differentiate into cancer-associated fibroblasts (CAFs), which in turn promote the tumor development of HNSCC [9,10,11,12,13].

To further characterize the role of EMT in tumor progression, we analyzed the expression of EMT markers ZEB2, SNAI2, and β-catenin in an experimental 3D tumor stroma. In addition to the role of Akt, Erk1/2 and mTOR pathways in tumor transition as well as CD24 and its influence on immune competence in the TME were analyzed. MSCs offer a well-characterized model to study the TME since they have been characterized with Janus-like capabilities as tumor suppressors as well as promoters [14,15]. In addition, 3D culture analysis and multicellular tumor spheroids of HNSCC have been employed for exploring and monitoring tumor differentiation and progression [16]. Analyzing stroma-tumor interactions in 3D systems is currently of growing interest to reproduce tumor mass architecture [17]. Using a hybrid 3D culture model offers a unique investigation to assess tumor transition, noting the heterogeneous cellularity of the TME [18]. In our study, we analyzed the effect of TME, EMT, and tumor–stroma interaction in a model of 3D stroma-tumor spheroids (STSs) using MSCs and the established tumor cell line SCC-040.

## 2. Materials and Methods

### 2.1. Cultivation of Human SCC-040 and Human Mesenchymal Stem Cells

SCC-040 (UICC: T2 N2) is an established human cell line from a stage 4 new primary oral squamous cell carcinoma in a 50-year-old Caucasian male (DSMZ-Germany, Braunschweig, Germany) [19,20,21]. SCC-040 was cultured in PCI medium consisting of Minimum Essential Medium Earle’s with 10% fetal bovine serum, 1% nonessential amino acids, and 1% penicillin streptomycin. Human MSC was obtained from Lonza, Basel, Switzerland (LOT-no. 0000602009) and cultured in MSC medium composed of DMEM, 10% fetal bovine serum, 1% nonessential amino acids, and 1% penicillin streptomycin. MSCs displayed classic morphologic characteristics as small, self-renewing, spindle-shaped cells [22,23], while SCC-040 displayed an adherent squamous cell monolayer as described by the manufacturer. All cell culture experiments were performed at 37 °C and 5% CO_2_. At 80–90% confluence, the cells were trypsinized, washed, counted (CASY model TT, Schärfe-System GmbH, Reutlingen, Germany), and prepared for further experiments.

### 2.2. Stroma-Tumor Spheroids (STSs)

A stock suspension of 1 × 10^6^ cells/mL was prepared according to the experimental requirements, and STSs were prepared according to the hanging drop method (Kramer Rohwedel Böhrnsen) [24]. To simulate stroma-rich and -poor tumors, cocultured STSs as well as control groups were created according to defined cell concentrations (Table 1). STSs were created with a content of 30,000 cells each.

With a 100 µL pipettor, 30 µL of the desired STS-experimental group was placed on the bottom of a 100 mm TC-treated cell culture dish. The dish was turned upside down to create “hanging droplets” [19]. A lid of a 60 mm cell culture dish filled with PBS was placed within the STS cultivation chamber to avoid evaporation. Incubation occurred at 37 °C and 5% CO_2_ for 48 h until STS formation was visible. MSCs were cultured in MSC medium, while SCC and cocultured STS were cultured in PCI medium. After 48 h, STS were flushed down, collected using a 1000 µL pipettor, and washed twice with PBS for further analysis [19].

### 2.3. Real-Time qPCR (RT-qPCR) Analysis of STS

RNA was isolated using a standardized RNA isolation kit (RNeasy Mini Kit, Qiagen, Hilden, Germany) according to the manufacturer’s recommendations. The concentration of RNA was determined by measuring the absorbance at 260 and 280 nm with a spectrophotometer (NanoDrop1000, PEQLAB, Erlangen, Germany). Samples of 1000 ng RNA were reverse-transcribed (iScript™ cDNA Synthesis Kit, Bio-Rad Laboratories, Hercules, CA, USA) according to the manufacturer’s recommendations. All primers were provided by Thermo Fisher Scientific (Waltham, MA, USA). They were specific for GAPDH (forward 5′- TCCTCCTGTTCGACAGTC-3′, reverse 5′- ATCTCGCTCCTGGAAGATGGT-3′, 310 bp, NM_002046.7); CD24 (forward 5′- GCACTGCTCCTACCCACG-3′, reverse 5′- GCAGAAGAGAGAGTGAGACCAC-3′, 181 bp, NM_013230.3); β-catenin (forward 5′- GAAACGGCTTTCAGTTGAGC-3′, reverse 5′- CTGGCCATATCCACCAGAGT-3′, 166 bp, NM_001904.4); SNAI2 (forward 5′- AAGCATTTCAACGCCTCCAAA-3′, reverse 5′- GGATCTCTGGTTGTGGTATGACA-3′, 118 bp, NM_003068.5); and ZEB2 (forward 5′- AGGAGCACATCAAGTACCGC-3′, reverse 5′- CCTGCTCCTTGGGTTAGCAT-3′, 154 bp, NM_014795.4). After establishing the optimum annealing temperature by means of gradient PCR (HOT FirePol^®^ DNA Polymerase, Solis Biodyne, Tartu, Estonia), quantification of the cDNA was performed by using a Bio-Rad myIQ real-time PCR detection system and Bio-Rad iQ SYBR Green Supermix. Initial denaturation and enzyme activation occurred at 95 °C for 3 min, followed by 40 cycles of denaturing at 95 °C for 15 s and annealing and extension at 64 °C for 30 s. No additional signals were detected for any PCR product. Negative controls were included.

### 2.4. Immunohistochemical Analysis (IHC) of Vimentin, Connexin 43, and Emmprin (CD147)

IHC staining was performed with antibodies specific for the following proteins: vimentin, connexin 43 (Cx43), and emmprin (CD147). After cultivation, STSs were collected, washed twice with PBS, and fixed in 4% formaldehyde overnight. After dehydration with a graded series of ethanols (50–100%), STSs were embedded in paraffin. We transferred 2 µm tissue sections onto microscope slides, which we prepared for further analysis. Following deparaffinization, epitope retrieval was performed with Target Retrieval Solution (Dako, Carpinteria, CA, USA) at pH 8.5 in a pressure cooker (121 °C 30 s, 90 °C 10 s, 16 mbar). The samples were cooled for 5 min in ice-cold dH₂O, followed by washing with PBS. A peroxidase blocking solution was applied and incubated for 17 min at room temperature (RT). Samples were washed, and bovine serum albumin (BSA) blocking solution (10% BSA in PBS) was applied for 1 h at RT. BSA was removed, and the following antibodies were applied (designation, dilution ratio in PBS, and company are given in parentheses): vimentin (SP20, 1:500_PBS_; Thermo Fisher Scientific, Waltham, MA, USA), connexin 43 (#3512, 1:50_PBS_; Cell Signaling Technology, Danvers, MA, USA), and emmprin (10E10, 1:144_PBS_; abcam, Cambridge, UK). Samples were stored at 4 °C overnight. Samples were washed, and a secondary antibody was applied. For vimentin and Cx43, anti-IHC-IgG (P0217, Dako, Carpinteria, CA, USA) was used in a dilution ratio of 1:100 (antibody diluent, Dako, Carpinteria, CA, USA). For emmprin, anti-IHC-IgG (ab6789, abcam, Cambridge, UK) was applied in a dilution ratio of 1:1000. Incubation occurred for 1 h at RT. Samples were washed, and samples were incubated with 3,3′-diaminobenzidine (K3468, Dako, Carpinteria, CA, USA) in the dark at RT. The reaction was stopped with dH₂O, and counterstaining was performed with hemalum (1:2 H_2_O; Merck, Darmstadt, Germany). Samples were embedded in Entellan’s new rapid mounting medium (Merck, Darmstadt, Germany). Analysis was performed with a Keyence BZ-X710 microscope (KEYENCE, Neu-Isenburg, Germany). Positive and negative controls were included. Negative controls were performed with nonspecific antibodies only.

### 2.5. Immunohistochemical Analysis (IHC) of E-Cadherin

Samples were deparaffinized, and epitope retrieval was performed as mentioned above, followed by 5 min in dH_2_O and washing with PBS. After peroxidase blocking, the antibody was applied (E-Cadherin #3195, 1:100_PBS_; Cell Signaling Technology, Danvers, MA, USA). After incubation overnight at 4 °C and washing, anti-IHC-IgG (414141F, Nichirei Biosciences Inc., Tokyo, Japan) was applied. Samples were washed and incubated with AEC substrate (BD Pharmingen, San Diego, CA, USA). Counterstaining was performed with hemalum, as explained above. Embedding was performed with Aquatex mounting medium (Merck, Darmstadt, Germany). Analysis was performed with a Keyence BZ-X710 microscope. Positive and negative controls were included. Negative controls were performed with non-specific antibodies only.

### 2.6. Akt Signaling Analysis

After collection, STSs were washed with PBS and lysed using a standardized cell lysis kit (PathScan^®^ Sandwich ELISA Lysis Buffer 1X, Cell Signaling Technology, Danvers, MA, USA). Protein concentration was determined using a Pierce™ BCA Protein Assay Kit (Thermo Fisher Scientific, Waltham, MA, USA) according to the manufacturer’s recommendations. Equal amounts of protein concentrations were used in a PathScan^®^ Akt Signaling Antibody Array Kit (Cell Signaling Technology, Danvers, MA, USA) for each kind of STS. Visualization occurred through LumiGLO^®^/Peroxide reagent and a chemiluminescent development folder (LAS-3000 Imaging System, GE Healthcare, Chalfont St. Giles, UK). Relative protein expression was analyzed via computer-assisted densitometry in relation to the provided standard. Positive as well as negative and background controls were always included.

### 2.7. Statistical Analysis

Statistical analysis was performed using GraphPad Prism (GraphPad Software Inc., La Jolla, CA, USA). Quantification of the RT-qPCR ratios was performed using the ΔCt method. Differences were analyzed and identified with the Kruskall–Wallis test followed by Dunn’s multiple comparisons test. Alpha was set to 0.05. Digital analysis of protein expression detected with IHC staining was performed via ImageJ software (Wayne Rasband, National Institute of Health/NIH, Bethesda, MD, USA). At least 13 fields of view (up to 30 fields of view) were analyzed per sample and per group. Analysis was performed via GraphPad Prism with the Kruskall–Wallis test and Dunn’s multiple comparisons test. Alpha was set to 0.05.

Relative protein expression concerning Akt signaling was determined via computer-assisted densitometric measurement. Results were analyzed with GraphPad Prism software via an ordinary one-way ANOVA and Tukey’s multiple comparisons test with alpha set to 0.05. All the data reported in this paper are part of the author’s doctoral thesis.

## 3. Results

### 3.1. Expression of EMT Marker Proteins, Connexin 43, and Emmprin in STS

**Figure 1:**
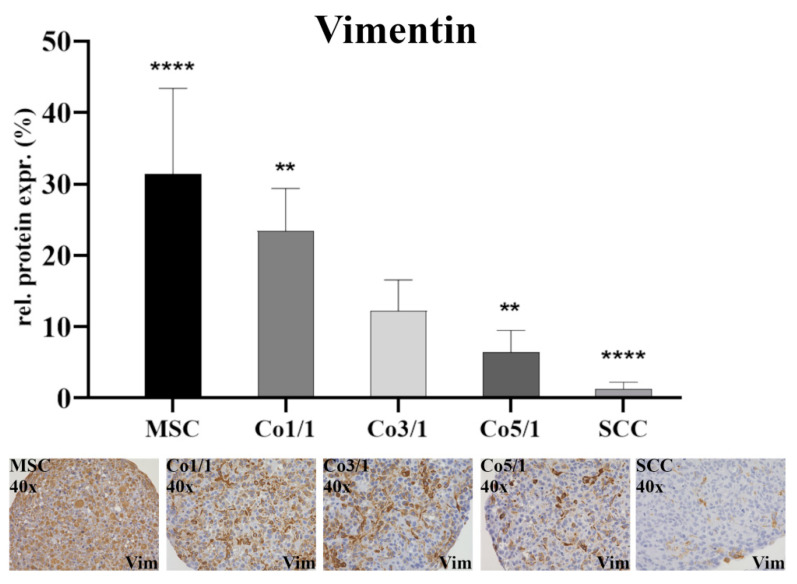
Immunohistochemical staining of vimentin in MSCs, Co1/1, Co3/1, Co5/1, and SCCs; magnification 40×. **** = *p* ≤ 0.0001, ** = *p* ≤ 0.01.

Protein expression was assessed via immunohistochemical staining of vimentin, connexin 43, E-cadherin, and emmprin. The immunostaining of vimentin (Figure 1) showed the highest expression in MSCs (*p* ≤ 0.0001). With an increased amount of tumor cells, the expression of vimentin dropped. SCCs showed the lowest expression of vimentin (*p* ≤ 0.0001). This continuous decrease could be observed in all samples, with Co3/1 (*p* = 0.0011) and Co5/1 (*p* = 0.0013) demonstrating a significantly higher or lower expression of vimentin, respectively.

**Figure 2:**
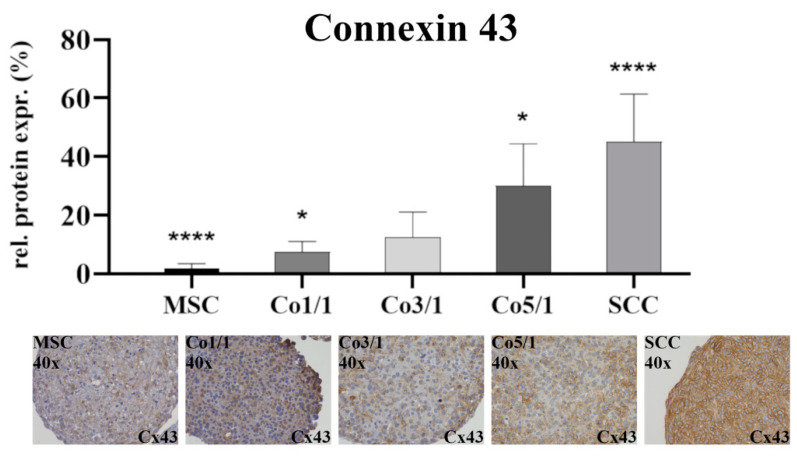
Immunohistochemical staining of connexin 43 in MSCs, Co1/1, Co3/1, Co5/1, and SCCs; magnification 40×. **** = *p* ≤ 0.0001, * = *p* ≤ 0.05.

Immunostaining of connexin 43 (Cx43) (Figure 2) showed the lowest expression in MSCs (*p* ≤ 0.0001). With an increased amount of tumor cells, the expression of Cx43 increased. SCCs showed the highest expression (*p* ≤ 0.0001). This continuous increase from low to high could be observed in all samples, with Co5/1 (*p* = 0.0224) and Co3/1 (*p* = 0.0023) showing additional significance.

**Figure 3:**
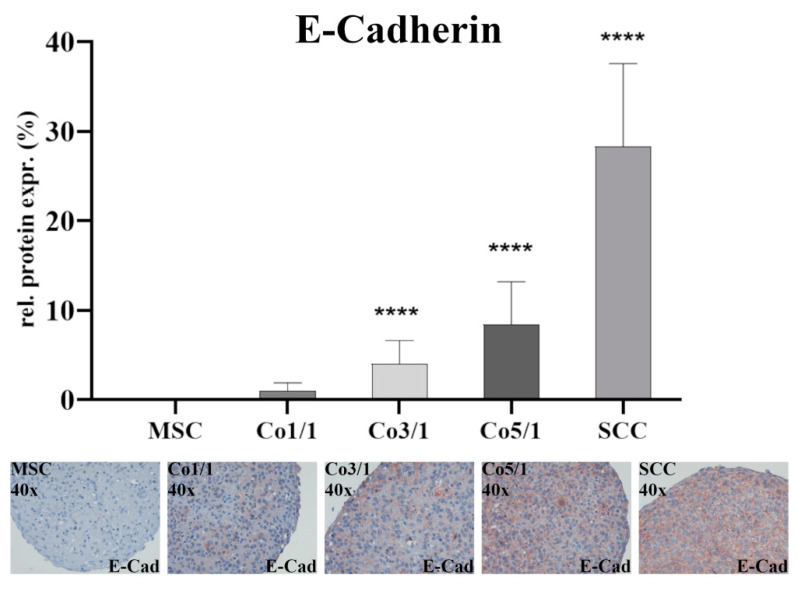
Immunohistochemical staining of E-cadherin in MSCs, Co 1/1, Co3/1, Co5/1, and SCCs; magnification 40×. **** = *p* ≤ 0.0001.

Immunostaining of E-cadherin (Figure 3) showed almost no expression in MSCs. The expression increased with an additional amount of tumor cells with the highest expression being observed in SCCs (*p* ≤ 0.0001). While no difference was found between MSCs and Co1/1, E-cadherin expression significantly increased in Co3/1 (*p* ≤ 0.0001) and Co5/1 (*p* ≤ 0.0001).

**Figure 4:**
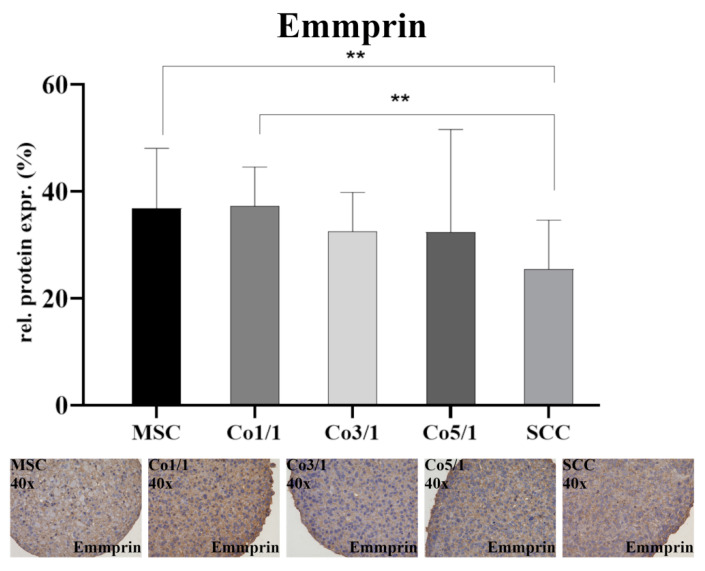
Immunohistochemical staining of emmprin in MSCs, Co1/1, Co3/1, Co5/1, and SCCs; magnification 40×. ** = *p* ≤ 0.01.

Emmprin was expressed in all samples (Figure 4). The expression of emmprin was significantly higher in MSCs (*p* = 0.0018) and Co1/1 (*p* = 0.0094) than in SCCs, which expressed the lowest amount of emmprin. Unlike vimentin, Cx43, and E-cadherin, an increased amount of tumor cells did not alter the expression of emmprin. No significant changes in emmprin expression were detected when comparing MSC, Co1/1, Co3/1, and Co5/1.

### 3.2. Expression of EMT Marker Genes and CD24 in STSs

**Figure 5:**
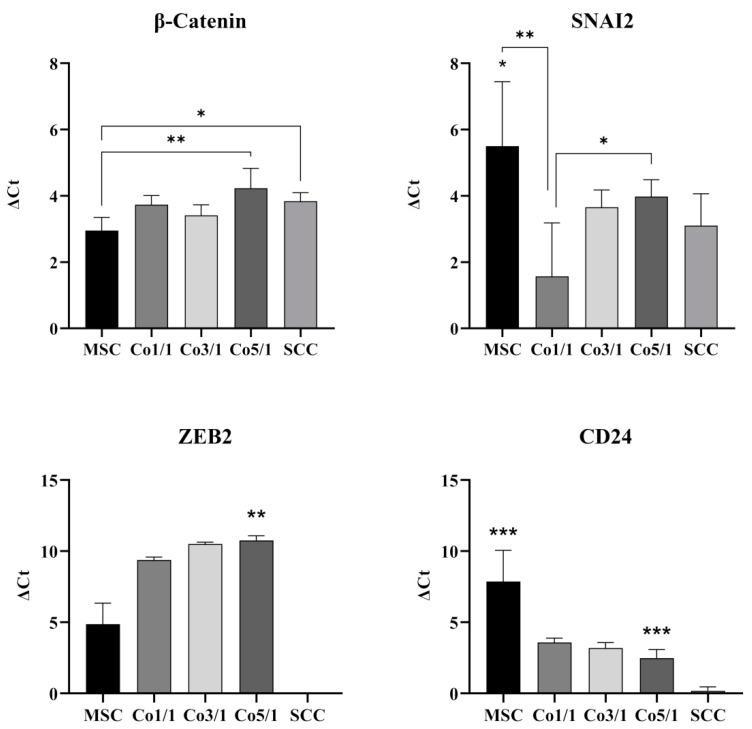
RT-qPCR analysis of β-catenin, SNAI2, ZEB2, and CD24 in MSCs, Co1/1, Co3/1, Co5/1, and SCCs (*** = *p* ≤ 0.01, ** = *p* ≤ 0.01, * = *p* ≤ 0.05).

Transcriptomic analyses of the gene expression profiles of EMT marker genes β-catenin, SNAI2, ZEB2, and CD24 were assessed via RT-qPCR (Figure 5). β-catenin was expressed in all samples. MSCs showed the lowest expression of β-catenin. However, an increase in tumor cells showed significantly higher expressions of β-catenin in SCCs (*p* = 0.0269) and Co5/1 (*p* = 0.0031) compared with MSCs. RT-qPCR revealed the highest expression of SNAI2 in MSC. Interestingly, an addition of tumor cells results in a decreased expression of SNAI2 (Co1/1, *p* = 0.0045). However, the expression increased with the amount of tumor cells added, reaching significant levels of expression in Co5/1 (*p* = 0.0352), which had the highest amount of tumor cells in all setups.

ZEB2 was expressed in all MSCs and MSC-tumor samples. The expression increased with an addition of tumor cells. However, SCCs alone did not express significant levels of ZEB2. The highest amount of ZEB2 expression was observed in Co5/1 (*p* = 0.0099). The expression of CD24 was also found to be very low in SCCs (*p* = 0.0004). MSCs revealed the highest expression of CD24 (*p* = 0.0003). Similar to SNAI2, the expression of CD24 dropped with the addition of tumor cells and reached its lowest point in SCCs.

### 3.3. Protein Expression of S6 Ribosomal Protein, mTOR, p70 S6 Kinase, and Erk 1/2 in STSs

**Figure 6:**
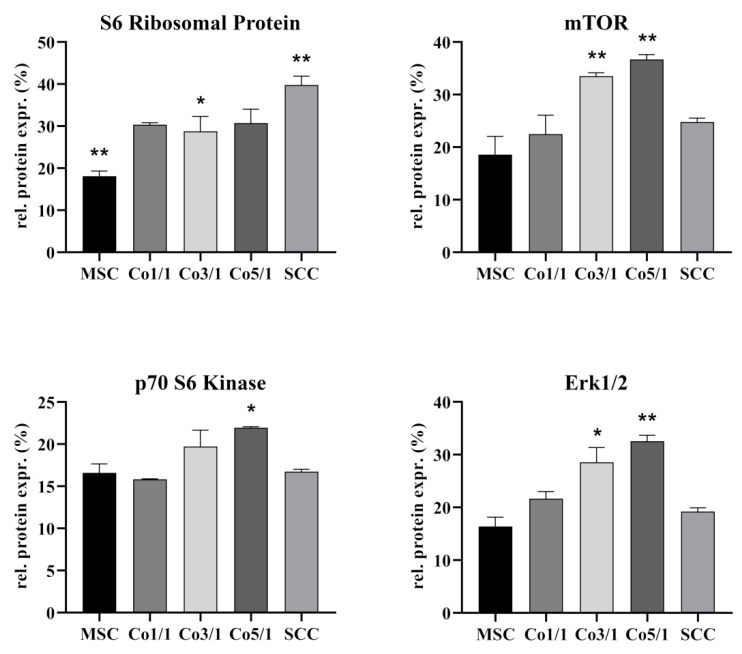
Protein level analysis of S6 ribosomal protein, mTOR, p70 S6 kinase, and Erk1/2 in MSCs, Co1/1, Co3/1, Co5/1, and SCCs (** = *p* ≤ 0.01, * = *p* ≤ 0.05).

To further characterize interactions between MSCs and SCCs, we analyzed the expression of S6 ribosomal protein (RP S6), mTOR, p70 S6 kinase (Thr421/Ser424), and Erk1/2 (Figure 6). MSCs expressed significantly less RP S6 (*p* = 0.0358) than all other tested groups, whereas SCCs showed the highest expression of RP S6 (*p* = 0.0016). Following this trend, the expression of RP S6 increased with the amount of tumor cells added to STSs, with Co3/1 expressing significantly more RP S6 than MSCs (*p* = 0.0358) but still significantly less than SCCs (*p* = 0.0319). MSCs expressed the lowest amount of mTOR. With the addition of tumor cells, the expression increased, demonstrating the highest rate in Co3/1 and Co5/1 (*p* = 0.007). No significance was observed between MSCs, SCCs, and Co1/1. Analyzing the expression of p70 S6 kinase, a similar trend was found, with Co5/1 demonstrating the highest significant increase (*p* = 0.016). Comparable to mTOR, the expression of Erk1/2 also increased with the addition of tumor cells. The highest expression was found in Co3/1 and Co5/1 (*p* = 0.0045). Comparable to the expression of the mTOR protein, the expression of Erk1/2 decreased in the SCCs with no MSCs present. No significance was observed between MSCs, SCCs, and Co1/1.

## 4. Discussion

The TME and tumor progression are influenced by tumor–stroma interactions. MSCs are part of the TME [15]. The chemoattractants and cytokines released as a consequence of tumor progression often lead to an inflammatory tumor setting, resulting in a migratory response of MSCs and their integration into the TME [15,25,26]. MSCs show a strong tropism for tumor-related inflammation, trying to restore tissue integrity even under high stress [14,25]. While some studies suggest a tumor-suppressive attribute of MSCs, others have indicated a tumor-promoting effect of stromal cells [14,15,27,28]. These Janus-like functions are crucial to the TME and often promote EMT-related tumor progression [15,29]. In our study, the expression of the EMT marker vimentin dropped in 3D stroma-tumor spheroids with an increased tumorous component. This finding is atypical for an EMT-mediated tumor progression, which would otherwise suggest an increasing expression of vimentin [30,31,32]. While vimentin is a marker of EMT and its expression was significantly higher in MSCs (*p* ≤ 0.0001), its expression can vary in different types of tumors and has been shown to be lower in HNSCC [33]. Similarly, the expression of E-cadherin is usually higher in epithelial tumors such as HNSCC [33,34]. In our study, a significantly low expression of E-cadherin in Co1/1 (*p* = 0.0384) aligned with a significantly higher expression of vimentin in Co1/1 (*p* = 0.0013), demonstrating opposing expression patterns. This stresses the codependent link between those two EMT marker proteins. However, it also demonstrates that EMT is influenced by individual TME attributes and can vary among tumor configurations. It is therefore interesting to determine the tumor turning point (TTP) in a codependent TME. The TME during tumor progression shows different stages and functional organization related to the model of tumor transition [35]. During this transition, cancer stem-like cells at the edges of the tumor display an advancing tumor frontier [36]. Interestingly, it was demonstrated that the transformation of the TME can result in the recruitment or re-education of healthy stromal cells, marking a turning point in cancer progression and mirroring cancer cell metabolism [37].

Therefore, the TTP marks a point during tumor–stroma communication where a small change in the TME may lead to a significant shift in tumor development, which ultimately contributes to the tumor’s progression and hinders recession. While SCCs alone did not express the EMT marker ZEB2, the combination of MSCs and SCCs suddenly resulted in an increasing expression of ZEB2, with the highest expression in Co5/1 (*p* = 0.0099). Similar alterations were found when looking at the expression of CD24. CD24 is known to be a surface marker of cancer stem cells, demonstrating a high ability to self-renew and enhance therapy resistance [38,39,40]. While SCCs did not show an increased expression of CD24, the introduction of small amounts of TME cells such as MSCs resulted in a significant increase in CD24 expression (Co5/1, *p* = 0.0003). SNAI2 also demonstrated an increase in expression correlating with an increasing tumor component in Co5/1 (*p* = 0.0352). SNAI2 is known to modulate and promote EMT as well as having antiapoptotic functions [41,42,43,44]. Similar to the expression of ZEB2 and CD24, β-catenin showed a significant increase in expression following MSC cocultivation in 3D spheroids (Co5/1, *p* = 0.0031). Comparable results on the codependence of MSCs and SCCs in the TME were found for the increasing expression of Akt-pathway proteins Erk1/2, p70 S6 kinase, and mTOR. Studies suggest that progressive TME changes occur within a rather short period of time [45,46]. In our study, small alterations in 3D coculture led to significant changes in genetic and protein expression in comparison with MSCs and SCCs alone. Especially mTOR and Erk1/2 are known as targets of anticancer drugs [47]. Upregulation of RP S6, mTOR, and Erk1/2 correlates with a cell cycle activation, which in turn promotes tumor proliferation, infiltration, and EMT [48,49,50,51]. Deregulation of mTOR at the TTP can lead to an increased expression of VEGF, contributing to invasiveness and proliferation [48,51]. Our findings indicate changes in mTOR, Erk1/2, and p70 S6 as well as genetic expression patterns at the transition between Co3/1 and Co5/1. The expression of Cx43 also supports a TTP between Co3/1 and Co5/1, with a significantly increased expression in Co5/1 (*p* = 0.0224). A TTP between Co3/1 and Co5/1 can also be assumed regarding ZEB2 and SNAI2 indicating a shift toward an EMT progression.

While different TTPs will have to be assumed for different tumors and individuals, they nonetheless would mark the threshold where tumor–stroma communication leads to a significant shift, which can ultimately contribute to the development of progression-related characteristics. Cell proliferation, growth, and maintenance of tissue homeostasis become affected and dysregulated. While the Ki67 antigen as well as proliferating cell nuclear antigen (PCNA) have been used to monitor changes in cell proliferation, the predictability of Ki67 and PCNA is influenced by internal and external factors and may vary in different tumors [52]. In addition, tumor biology, TTP as well as vascularization, proliferation, EMT, and extracellular matrix progression are influenced by external factors including VEGF, TNF, and TGF [53,54]. Although SCC-040 (UICC: T2 N2) is an established human cell line of a stage four new primary oral squamous cell carcinoma, its only use represents a limitation in this study. Established cell lines are commonly used as a model for tumor biology. However, it is an open question how to best apply or choose the available cell lines to represent cancer biology. Experimental follow-up work and multiomics analyses on established cell lines such as SCC-040 or CAL27 will provide further evidence to support or dismiss the use of an established cancer cell line [55], especially because tissue/cell type mislabeling may result in cell line misidentification commonly due to cross-contamination [56].

Continuous cell–cell interactions between tumor cells and surrounding stromal cells have been found to lead to EMT, resistance, and immune escape [57,58,59]. TME-mediated resistance and immune escape are linked to the heterogeneity within the TME and its evolving conditions [6]. MSCs have been shown to contribute to invasive characteristics of the TME [13]. In addition, a change in CD24 expression, as observed in our study, is known to be indicative of a cancer stem cell shift able to promote EMT and tumor progression. Here, MSCs exert their Janus-like influence on self-renewal and therapy resistance [60] by causing changes in the gene expression profile of tumor cells [36,39,40,61]. Using 3D STS analysis, it is possible to complement previous studies in the assessment of the TTP, especially since the development of stroma-tumor spheroids offers an opportunity for preclinical high-throughput analysis [62,63].

## Figures and Tables

**Table 1:** Proportions of cell types used to create different STSs as mono- and cocultures.

Stroma-Tumor Spheroids (STSs)	SCC-040	MSC
MSC (control)	0	6 × 10^5^
Co1/1 (1:1 coculture)	3 × 10^5^	3 × 10^5^
Co3/1 (3:1 coculture)	4.5 × 10^5^	1.5 × 10^5^
Co5/1 (5:1 coculture)	5 × 10^5^	1 × 10^5^
SCC (control)	6 × 10^5^	0

## Data Availability

Not applicable.
